# Anti-Glycation Properties of Zinc-Enriched *Arthrospira platensis* (Spirulina) Contribute to Prevention of Metaflammation in a Diet-Induced Obese Mouse Model

**DOI:** 10.3390/nu16040552

**Published:** 2024-02-17

**Authors:** Eleonora Aimaretti, Elisa Porchietto, Giacomo Mantegazza, Giorgio Gargari, Debora Collotta, Giacomo Einaudi, Gustavo Ferreira Alves, Enrica Marzani, Alessandro Algeri, Federica Dal Bello, Manuela Aragno, Carlo Cifani, Simone Guglielmetti, Raffaella Mastrocola, Massimo Collino

**Affiliations:** 1 Department of Clinical and Biological Sciences, University of Turin, 10125 Turin, Italy; eleonora.aimaretti@unito.it (E.A.); manuela.aragno@unito.it (M.A.); raffaella.mastrocola@unito.it (R.M.); 2 Pharmacology Unit, School of Pharmacy, University of Camerino, 62032 Camerino, Italy; elisa.porchietto@unicam.it (E.P.); giacomo.einaudi@unicam.it (G.E.); gustavo.ferreiraalves@unito.it (G.F.A.); carlo.cifani@unicam.it (C.C.); 3 Department of Food, Environmental and Nutritional Sciences (DeFENS), University of Milan, 20133 Milan, Italy; giacomo.mantegazza@unimi.it (G.M.); giorgio.gargari@unimi.it (G.G.); 4 Department of Neurosciences “Rita Levi Montalcini”, University of Turin, 10125 Turin, Italy; debora.collotta@unito.it (D.C.); enrica.marzani@unito.it (E.M.); 5 Italian Union of Biological Spirulin (Unione Spirulina Biologica Italiana, USBI), Curtatone (Mantova), 46010 Mantova, Italy; info@unionespirulina.it; 6 Department of Molecular Biotechnology and Health Sciences, University of Turin, 10126 Turin, Italy; federica.dalbello@unito.it; 7 Department of Biotechnology and Biosciences (BtBs), University of Milano-Bicocca, 20126 Milan, Italy

**Keywords:** advanced glycation end products (AGEs), *Arthrospira platensis* (spirulina), zinc supplementation, metaflammation, glyoxalase-1 (GLO1), receptor for AGEs (RAGE)

## Abstract

Advanced glycation end products (AGEs) exert a key pathogenic role in the development of obesity and insulin resistance. Thanks to its abundance in bioactive compounds, the microalga *Arthrospira platensis* (spirulina, SP) is proposed as a nutritional supplement. Here, we investigated the potential anti-glycating properties of SP enriched with zinc (Zn-SP) and the following impact on diet-induced metabolic derangements. Thirty male C57Bl6 mice were fed a standard diet (SD) or a high-fat high-sugar diet (HFHS) for 12 weeks, and a subgroup of HFHS mice received 350 mg/kg Zn-SP three times a week. A HFHS diet induced obesity and glucose intolerance and increased plasma levels of pro-inflammatory cytokines and transaminases. Zn-SP administration restored glucose homeostasis and reduced hepatic dysfunction and systemic inflammation. In the liver of HFHS mice, a robust accumulation of AGEs was detected, paralleled by increased expression of the main AGE receptor (RAGE) and depletion of glyoxalase-1, whereas Zn-SP administration efficiently prevented these alterations reducing local pro-inflammatory responses. 16S rRNA gene profiling of feces and ileum content revealed altered bacterial community structure in HFHS mice compared to both SD and HFHS + Zn-SP groups. Overall, our study demonstrates relevant anti-glycation properties of Zn-SP which contribute to preventing AGE production and/or stimulate AGE detoxification, leading to the improvement of diet-related dysbiosis and metabolic derangements.

## 1. Introduction

The current pandemic in obesity and correlated metabolic diseases, including type 2 diabetes and its complications, has become a worldwide emergency due to its massive socioeconomical burden [1,2]. Substantial changes in nutritional habits have occurred in industrialized as well as developing countries in recent decades, consisting in a hugely increased consumption of sugars and fats and ultra-processed foods, which is the main cause for this process [1]. A deeper understanding of the molecular pathways underlying the onset and development of obesity and insulin resistance is the point of departure for the formulation of pharmacological and nutraceutical tools able to prevent or relieve metabolic diseases [3,4,5].

A detrimental consequence of the modern nutrition is the progressive tissue accumulation of advanced glycation end products (AGEs), toxic compounds deriving from the reaction between reducing sugars or fatty acids and the amino group of proteins leading to dysfunctional undegradable glycated proteins [6,7]. AGEs can be of exogenous origin when ingested through the consumption of ultra-processed foods and foods cooked at high temperature, or they can be endogenously produced as a consequence of high-sugar and high-fat diets [8]. We previously demonstrated in animal models of diet-induced obesity the direct interference of either exogenous or endogenous AGEs on different metabolic pathways contributing to alterations of lipid metabolism and to the onset of a low-grade chronic inflammation and insulin resistance, comprehensively known as metaflammation [9,10,11].

Besides modifications in food processing and healthier nutritional approaches, the supplementation with several bioactive compounds of natural origin has been reported to exert anti-glycation effects, thus improving many of the metaflammation aspects [12,13]. Consistently, we and others have demonstrated that the inhibition of AGE production through supplementation with the vitamin B6 analog pyridoxamine, which is able to quench the dycarbonil precursors of AGEs, was effective in the amelioration of obesity, lipid metabolism, inflammation, as well as insulin resistance [9,10,14,15].

Among the number of beneficial organic supplements able to reduce inflammation and oxidative stress, the microalga *Arthrospira platensis*, also known as spirulina, has gained much attention as an excellent functional food supplement because of its richness in nutritional components [16,17]. Spirulina is a spiral blue-green microalga belonging to the Cyanobacteriota phylum. It is a remarkable source of high-quality protein with nearly all essential amino acids, vitamins, minerals, fibers, and antioxidants agents including phycocyanins, carotenoids, tocopherols, and phenolic compounds [18,19,20,21]. In addition, spirulina contains about 8% of lipids mainly represented by essential poly unsaturated fatty acids γ-linolenic acid, α-linoleic acid, eicosapentaenoic acid (EPA), and docosahexaenoic acid (DHA) [19,20]. For its high nutritional value, spirulina is actually considered a superfood, able to effectively improve antioxidant status and lipid metabolism, and even protecting against cancer development [18,19,20]. Although spirulina is found to be effective in improving metabolic derangements in animal studies [22,23,24], results on its effects on glycemic control and inflammation in humans are still inconclusive [18,24,25], suggesting the need of a deeper investigation on the mechanisms of action of the different bioactive ingredients [25]. However, due to the complex mixture of several nutritional components providing antioxidant and anti-inflammatory effects and modulatory action in lipid metabolism and gut microbiota, it is not possible to discriminate which are the main individual contributors to the amelioration of metabolic inflammation. Interestingly, among the multiple mechanisms of protection exerted by the different spirulina components, no literature data are available about its possible effect on glycation. Thus, in the present study, we aimed to investigate whether the supplementation with lyophilized spirulina extract is able to counteract the metaflammation induced by a high-fat high-sugar diet in mice and whether this is mediated by the prevention of the hepatic accumulation of AGEs. 

A peculiar characteristic of spirulina cells is their natural ability to accumulate metals during growth, which is essential for the use of spirulina biomass as a useful dietary supplement. Zinc is one of the main essential components among the trace elements used to cultivate this cyanobacterium, and it is one with the highest efficiency of accumulation by spirulina [26]. Zinc has an antioxidant potential through the nonenzymatic stabilization of biomembrane and biostructures and recent findings indicate a direct relationship between low zinc levels, greater body fat content, and insulin resistance [27]. Obese subjects have a higher risk of suffering from zinc deficiency when compared to lean individuals, and an improvement in their zinc status has been demonstrated to ameliorate their insulin sensitivity [28].

Thus, we decided to test here a spirulina extract enriched in zinc (zinc-enriched spirulina, Zn-SP), to possibly strengthen its antioxidant activity and protection against diet-induced metabolic derangements.

## 2. Materials and Methods

### 2.1. Zinc-Enriched Spirulina (Zn-SP)

The spirulina used in the experiment comes from an organic cultivation in Italy (Unione Spirulina Biologica Italiana USBI, Guidizzolo, Italy) and is obtained by filtration from tanks in greenhouses, where the culture can be controlled and confined. By enriching the culture medium at the end of the growth period, it was possible to obtain higher levels of zinc than those found in commercially available dried spirulina. The zinc enrichment was obtained through the depotentiation of other antagonistic ions present in the growth medium, thereby promoting the assimilation of the target element, in keeping with standard procedures that have been demonstrated to allow Zn-SP growth with no toxic effect and minimal increase in stress markers [29]. The concentration of zinc contained in the alga was determined using the Inductively Coupled Plasma–Optical Emission Spectroscopy (ICP-OES) technique. A volume of 20 mL of the culture medium was taken and filtered through a preweighed “Millipore” cellulose filter with a 0.45 µM porosity. After drying for 24 h at 48 °C, the filter was reweighed and mineralized for 30 min at 95 °C with 1.5 mL of sulfuric acid and 1.5 mL of concentrated nitric acid. After mineralization, the solution was filtered, made up to 50 mL with distilled water, and analyzed using an ICP-OES PerkinElmer Optima 8300 (Perkin Elmer Italia, Milan, Italy). Zn-SP nutritional formulation is reported in Table 1.

### 2.2. Animal Model

The present experimental procedures were approved by the local Animal Use and Care Committee and the Ministry of Health (approval no. 855/PR Ministry of Health, Rome, Italy) in accordance with the European Directive 2010/63/EU on the protection of animals used for scientific purposes as well as the Guide for the Care and Use of Laboratory Animals. Thirty-three-week old male mice C57BL/6 (Envigo RMS Srl, San Pietro al Natisone, UD, Italy) were maintained in conventional housing conditions in a controlled environment (25 ± 2 °C), 12/12 h light/dark cycle, with ad libitum access to food and water. After the acclimatation week, mice were randomly divided into one of the two dietary regimens: standard diet (SD) (3.85 kcal/g, D12450K, *n* = 10) and high-fat high-sugar diet (HFHS) (5.56 kcal/g, D12331 *n* = 20) for 12 weeks. Both diets were purchased from ssniff^®^ company (Soest, D-59494, Germany). After 5 weeks of dietary intervention, 10 mice from the HFHS-fed groups received Zn-SP at the dosage of 350 mg/kg trice a week. The dose of spirulina was selected according to previous in vivo protocols [30,31]. The enrichment in zinc in our Zn-SP formulation corresponded to a daily intake of zinc around 0.3 mg/kg b.w., which is largely lower than the doses reported to evoke toxicity in mice [32]. At the end of the experimental procedure, mice were put in individual metabolic cages for 12 h in fasting condition, to collect feces samples. Mice were then subjected to euthanasia with isoflurane (IsoFlo, Abbott Laboratories, North Chicago, IL, USA). Blood was collected through cardiac puncture in microtubes containing 5% ethylene-diamine-tetraacetic acid (EDTA) and then centrifuged at 10,000× *g* at room temperature to obtain plasma which was directly snap-frozen. Liver samples and ileum content were collected in tubes and snap-frozen in liquid nitrogen. Samples were then stored at −80 °C until use.

### 2.3. Oral Glucose Tolerance Test

After 16 h without food but free access to tap water, an oral glucose tolerance test (OGTT) was performed [33]. Specifically, mice were administered orally a 30% D-glucose solution (2 g/kg). Blood was collected from the saphenous vein prior to glucose administration and at different timepoints (15, 30, 60, 120 min), and glucose concentration was measured with Accu-check Aviva glucometer (Roche Diabetes Care Italy S.p.A, Monza, Italy), which provides suitable approximation of plasma glucose levels.

### 2.4. Blood Insulin

Plasma insulin level was detected by an enzyme-like immunosorbent assay kit ELISA (#EZRMI-13K, Millipore Corp., Billerica, MA, USA).

### 2.5. Hepatic Transaminases

Commercially available FAR Diagnostic kits (Verona, Italy) were utilized to colorimetrically quantify plasmatic levels of alanine aminotransferase ALT (#7018) and aspartate aminotransaminase AST (#7036), according to the manufacturer’s instructions.

### 2.6. Cytokines Immunoassay

Plasma levels of the inflammatory cytokines, interleukin-6 (IL-6), interleukin-1β (IL-1β), and interferon gamma (IFN-γ) were measured using an ELISA assay, as previously reported [33], following the manufacturer’s instructions. 

### 2.7. Liver Tissue Extraction

Hepatic proteins were extracted as previously reported [10,33]. To obtain total protein extraction (about 50 mg of tissue) liver samples were homogenized with a lysis buffer, containing protease and phosphatase inhibitors, and centrifuged at 13,000× *g* for 25 min at 4 °C. Supernatants were saved at −80 °C until use. To obtain cytosolic and nuclear proteins, liver tissue was homogenized at 10% (*w*/*v*) in a buffer and centrifuged at 1000× *g* for 5 min at 4 °C. Supernatants were then further centrifuged at 10,000× *g* at 4 °C for 40 min to isolate the cytosolic fraction. The pellets containing the intact nuclei were resuspended in an extraction buffer and incubated on ice for 30 min for high salt extraction, followed by centrifugation at 15,000× *g* for 20 min at 4 °C. The resulting supernatants were collected and stored at −80 °C until use. Pierce™ BCA Protein Assay Kit (Thermo Fisher Scientific, Waltham, MA, USA) was selected to assay protein content.

### 2.8. Advanced Glycation End Product (AGE) Quantification

Free Nε-carboxymethyl-lysine (CML) and Nε-carboxyethyl-lysine (CEL), the most studied markers of AGE accumulation in tissues, were assessed on total liver extracts (*n* = 7/10 per group) after a hydrolyzing step with 0.6 M trichloroacetic acid (C2HCl3O2) and 6 M hydrochloric acid (HCl) for 12 h at 60 °C, by means of the liquid chromatography mass spectrometry (LC-MS) as previously described [33]. The chromatographic separation was performed in an UltiMate™ 3000 HPLC system (Dionex, Milan, Italy) provided by a high-resolution LTQ Orbitrap mass spectrometer (Thermo Scientific, Rodano, Italy) with an atmospheric pressure interface and an electrospray ionization (ESI) source. Liver extracts were processed through a Phenomenex Synergi reverse-phase C18 column (dimensions: 150 × 2.1 mm, particle size: 3 μm) at a flow rate of 200 μL/min. The composition of the gradient mobile phase was as follows: 95/5 to 40/60 in 25 min, 5 mM heptafluorobutanoic acid/acetonitrile. The monitored protonated molecular ions were 205.1188 *m*/*z* for CML and 379.2094 *m*/*z* for CEL. Calibration data made with CML and CEL were used for quantitative determination of the sample’s analytes. 

### 2.9. Western Blotting Analysis

Amounts of liver extracts containing 50 µg of proteins were separated by SDS-PAGE and electrotransferred to PVDF membranes (GE10600023 Amersham™ Hybond^®®^ P Western blotting membranes, PVDF; Merck KGaA, Darmstadt, Germany), as previously described [33]. Membranes were then incubated with primary antibodies (diluted 1:1000, according to producer instruction), listed in Supplementary Table S1, diluted in TBS containing 2% nonfat dry milk and 0.05% Tween, followed by incubation with appropriated horseradish peroxidase (HRP)-conjugated secondary antibodies (7074S, Anti-rabbit IgG, HRP-linked Antibody, 7076S Anti-rabbit IgG, HRP-linked Antibody, Cell Signaling Technology, Inc., Denver, MA, USA) diluted in TBS containing 2% nonfat dry milk and 0.05% Tween (antibody dilution 1:5000 or 1:10,000). Chemiluminescence images of protein bands were detected using the Clarity Western ECL substrate (Bio-Rad) and the ChemiDoc™ Imaging System (Bio-Rad Laboratories, Hercules, CA, USA) and quantified by densitometric image analysis software (Image Lab Version 6.0.1; Bio-Rad). Results were normalized to the densitometric values of internal loading controls, including GAPDH bands for total and cytosolic extracts, and histone H3 bands for nuclear extracts, and then expressed as fold of standard-diet-fed mice values.

### 2.10. Glyoxalase-1 (Glo-1) Activity Assay

The enzymatic activity of Glo-1 was measured in mouse liver total protein extracts by spectrophotometry using the method of McLellan et al. [34], as previously described [33]. Specifically, an assay mixture (final volume 200 μL) containing 8 mM methylglyoxal (MG) and 2 mM GSH in a sodium phosphate buffer (50 mM, pH 7.4) was incubated at 37 °C for 10 min to reach the equilibration of hemithioacetal formation before the addition of the protein extract (20–30 μg). The increase in absorbance at 240 nm as a consequence of the formation of S-D-lactoylglutathione, by means of a microplate spectrophotometer (EnSight (PerkinElmer, Waltham, MA, USA), was monitored as a measure of Glo-1 activity.

### 2.11. Glutathione Assay

Reduced and total glutathione were assayed spectrophotometrically (412 nm) in liver total homogenates according to the method of Akerboom et al. [35]. Briefly, the two forms of glutathione GSH and GSSG were determined by means of a kinetic assay: the catalytic amounts of GSH or GSSG and glutathione reductase generate a continuous reduction of 5,5′-dithiobis (2-nitrobenzoic acid, DTNB) by nicotinamide adenine dinucleotide phosphate (NADPH). The ratio of GSSG to GSH form of glutathione has been calculated as a direct marker of oxidative stress. 

### 2.12. Microbiota Composition Analysis

The bacterial community structure was investigated using metataxonomics on fecal and ileum samples collected from mice at the end of the 12-week experiment. DNA extraction was performed using the QIAsymphony PowerFecal Pro Kit (Qiagen, Hilden, Germany) following the manufacturer’s instructions. Subsequently, 16S rRNA gene profiling was conducted as previously described [36,37]. In brief, the NovaSeq 6000 platform with 2 × 250 bp sequencing (NovaSeq 6000 SP Reagent Kit, 500 cycles, Illumina Inc., San Diego, CA, USA) was utilized to sequence the 16S rRNA gene amplicons, which covered the V3 and V4 variable regions obtained with primers 341F (5′-CCTACGGGNGGCWGCAG-3′) and 805R (5′-GACTACHVGGGTATCTAATCC-3′) (LC Sciences, Houston, TX, USA). The generated sequencing reads were processed using the bioinformatic pipeline Quantitative Insights Into Microbial Ecology (QIIME) 2 version 2022.2, employing the Divisive Amplicon Denoising Algorithm (DADA2) [38]. We measured intra-sample biodiversity (α-diversity) with four different indexes: observed features, as a measure of richness; Shannon’s entropy, which considers both evenness and richness; Pielou’s index, as a measure of evenness; and Faith’s phylogenetic diversity (Faith P.D.), which also considers the phylogenetic distances. The intersample diversity (beta-diversity) of the fecal microbiota composition was assessed calculating the weighted UniFrac distances plotted with a principal-coordinate analysis (PCoA). Taxonomic assignment to amplicon sequence variants (ASVs), clustered at 97% similarity, was performed using the Greengenes database v. 13_8. The metataxonomic data used in this study are available as FASTQ files in the European Nucleotide Archive (ENA) of the European Bioinformatics Institute, with the accession code PRJEB68206.

### 2.13. Statistical Analysis

Data distribution was evaluated with Shapiro–Wilk test. Statistical differences within the experimental groups were assessed with one-way ANOVA, followed by Bonferroni’s post hoc test. Data were expressed as mean ± SEM (standard error of the mean). GraphPad Prism 7.0 software package was used for the data analyses (GraphPad Software 7.0, San Diego, CA, USA). The threshold for statistical significance was established at *p* < 0.05. Statistical analyses on metataxonomic data were conducted using the R programming language (version 3.4.2). Taxa exhibiting differential abundance between groups were identified using the Mann–Whitney test applied to read abundances that underwent centered log-ratio (CLR) transformation. Furthermore, correlation analyses were performed, and Kendall’s τ rank correlation coefficient was calculated to assess the relationships within the data.

### 2.14. Chemical Reagents

If not differently stated, all analytical compounds were obtained from Sigma-Aldrich (Saint Louis, MO, USA).

## 3. Results

### 3.1. Effects of Dietary Manipulation and Zn-SP Supplementation on Systemic Metabolic Parameters

Twelve weeks of HFHS diet induced overt obesity in mice, leading to a final body weight double that of SD-fed mice (Table 2). As expected, calorie intake was significantly higher in mice fed the HFHS diet compared to SD mice, with no differences in daily food intake. 

**Figure 1:**
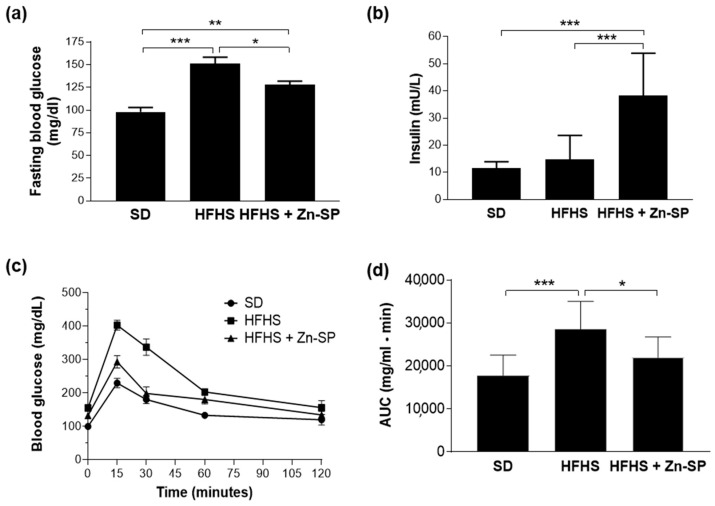
Glucose homeostasis. Mice were exposed to SD or HFHS diet for 12 weeks (SD *n* = 10–HFHS *n* = 20). After 5 weeks of dietary intervention, 10 mice from the HFHS group received Zn-SP (350 mg/kg p.o.) three times a week. After an over-night fasting, fasting blood glucose and insulin were measured ((**a**) and (**b**), respectively) and an OGTT (oral glucose tolerance test) was performed (**c**) and area under the curve (AUC) was calculated (**d**). All the data are expressed as mean ± SEM. Statistical significance: * *p* < 0.05; ** *p* < 0.01; *** *p* < 0.001. SD: standard diet; HFHS: high-fat high-sugar diet; HFHS + Zn-SP: HFHS + zinc-enriched spirulina.

The supplementation with Zn-SP to HFHS mice did not interfere with body weight gain nor with food and calorie intake. Interestingly, after 12 weeks of HFHS diet, mice displayed significantly increased fasting blood glucose compared to SD mice, with no difference in plasma insulin levels (Figure 1). HFHS mice also developed markedly impaired glucose tolerance, as shown by glycemic curves after glucose loading during OGTT and confirmed by significant differences in AUC values between HFHS mice and SD mice. Interestingly, Zn-SP administration to HFHS mice resulted in increased insulin release, which was associated with significant improvement in fasting blood glucose level and glucose tolerance.

### 3.2. Zn-SP Prevents AGE Accumulation in Liver

**Figure 2:**
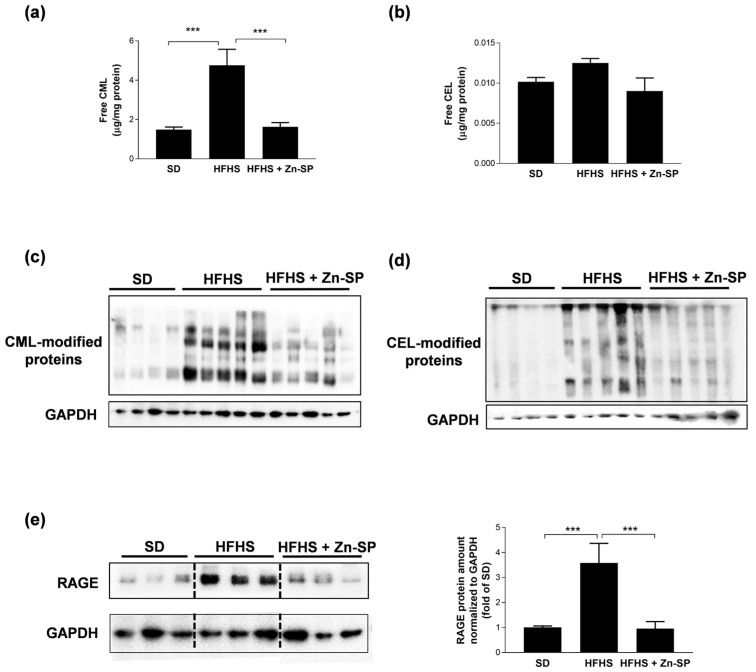
Hepatic AGE accumulation and RAGE expression. CML (**a**) and CEL (**b**) were analyzed by HPLC/MS and the results were normalized to total protein concentrations. Western blotting analysis for CML-modified proteins (**c**), CEL-modified proteins (**d**), and RAGE (**e**). Densitometric analyses of the bands were normalized to corresponding GAPDH density. Data are expressed as mean ± SEM of 4–5 mice per group. Statistical significance: *** *p* < 0.001. SD: standard diet; HFHS: high-fat high-sugar diet; HFHS + Zn-SP: HFHS + zinc-enriched spirulina; CML: carboxymethyl-lysine; CEL: carboxyethyl-lysine; RAGE: receptor for advanced glycation end products.

Since a well-known consequence of hyperglycemia is the production and tissue accumulation of AGEs, we wondered whether chronic Zn-SP administration was able to prevent AGE formation and protein glycation. We analyzed two major AGEs in mice livers, CML and CEL, both in their free (Figure 2a and Figure 2b, respectively) and protein-bound forms (Figure 2c,d). HFHS-fed mice accumulated markedly higher amounts of free CML and CML-modified proteins in the liver than SD-fed mice. Similarly, free CEL and CEL-modified proteins were increased in the livers of HFHS-fed mice, although free CEL modifications did not reach statistical significance. Most notably, when HFHS mice were administered with Zn-SP, both free and protein-bound CML and CEL were maintained at levels similar to those recorded in SD mice. AGE accumulation in the liver was paralleled by a massive increase in the protein expression of the main AGE receptor, RAGE (Figure 2e). The local hyperexpression of RAGE induced by the HFHS diet was completely abolished when mice were supplemented with Zn-SP. 

### 3.3. Zn-SP Enhances Antioxidant and Anti-Glycation Defenses in Liver

**Figure 3:**
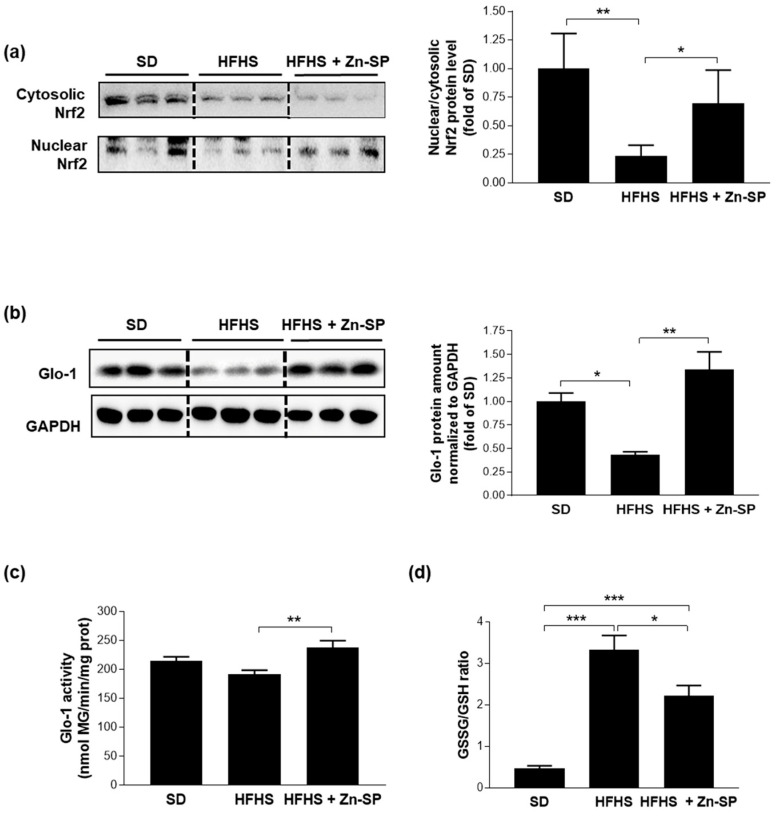
AGE detoxification. Western blotting analysis for Nrf2 nuclear translocation (**a**) and Glo-1 (**b**). Glo-1 enzymatic activity (**c**) and reduced and total glutathione (**d**) were assayed spectrophotometrically. Statistical significance: * *p* < 0.05; ** *p* < 0.01; *** *p* < 0.001. SD: standard diet; HFHS: high-fat high-sugar diet; HFHS + Zn-SP: HFHS + zinc-enriched spirulina; Nrf2: nuclear factor erythroid-related factor 2; GLO-1: glyoxalase-1.

Since the production of AGEs is enhanced in the presence of oxidative stress and, contrarily, is counteracted by physiologically active antioxidant and anti-glycation systems, we analyzed in liver homogenates the activation of Nrf2, the master transcription factor responsible for the expression of antioxidant enzymes, and Glo-1, the main AGE-detoxifying enzyme. We here observed that both the cytosolic and the nuclear protein level of Nrf2 in HFHS mice were downregulated compared to SD mice with lower nuclear-to-cytosolic protein level ratio, thus suggesting reduced expression and transcriptional activity of Nrf2 induced by the hypercaloric diet (Figure 3a). Accordingly, the protein level of Glo-1 was markedly downregulated in the liver of HFHS mice (Figure 3b). The corresponding enzymatic activity of Glo-1 was instead only slightly reduced compared to SD mice (Figure 3c), suggesting that the detoxifying activity of Glo-1 fails to be upregulated in the presence of excessive AGE accumulation. Moreover, we detected an impaired ratio between oxidized-to-reduced glutathione following HFHS exposure (Figure 3d), which agrees with the previous findings on impaired Nrf2 activation and Glo-1 enzymatic activity. In fact, GSH is the essential co-factor for Glo-1 activity, and the expression of enzymes recycling GSH is regulated by Nrf2. Interestingly, when Zn-SP was chronically administered to HFHS mice, the hepatic nuclear translocation of Nrf2 was restored, leading to a markedly upregulated expression of Glo-1 protein paralleled by enhanced enzymatic activity and reduction in the GSSG/GSH ratio.

### 3.4. Zn-SP Inhibits Diet-Induced Activation of Pro-Inflammatory Signaling in Liver

**Figure 4:**
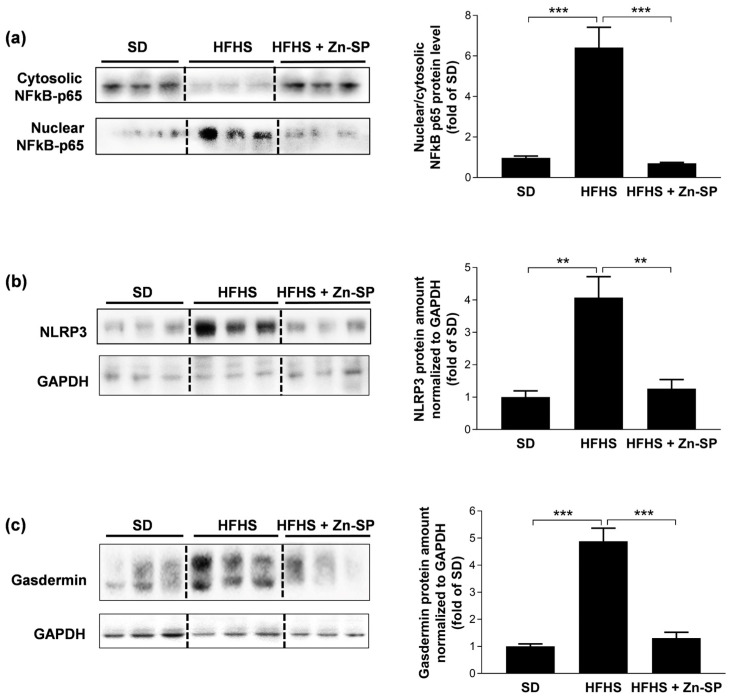
NFκB and NLRP3 inflammasome activation. Western blotting analysis for NFκB p65 nuclear translocation (**a**), NLRP3 (**b**), and gasdermin (**c**). Data are expressed as mean ± SEM of 4–5 mice per group. Statistical significance: ** *p* < 0.01; *** *p* < 0.001. SD: standard diet; HFHS: high-fat high-sugar diet; HFHS + Zn-SP: HFHS + zinc-enriched spirulina; NFκB: nuclear factor κ B; NLRP3: NLR Family Pyrin Domain Containing 3.

The AGE-RAGE binding is known to stimulate intracellular signal transduction leading to the activation of selective pro-inflammatory cascades. Here, we documented that HFHS-induced AGE accumulation in the liver was associated with a marked increase in the nuclear translocation of the p65 subunit of the NFκB signaling pathway (Figure 4a), as well as in the expression of the main proteins involved in the formation of the NLRP3 inflammasome complex, namely NLRP3 (Figure 4b) and gasdermin (Figure 4c). In contrast, the HFHS-induced activation of both NFκB and NLRP3 inflammasome cascades in the liver was drastically reduced by chronic Zn-SP administration.

### 3.5. Zn-SP Supplementation Counteracts Diet-Induced Increases in Blood Markers of Inflammation and Liver Injury

**Figure 5:**
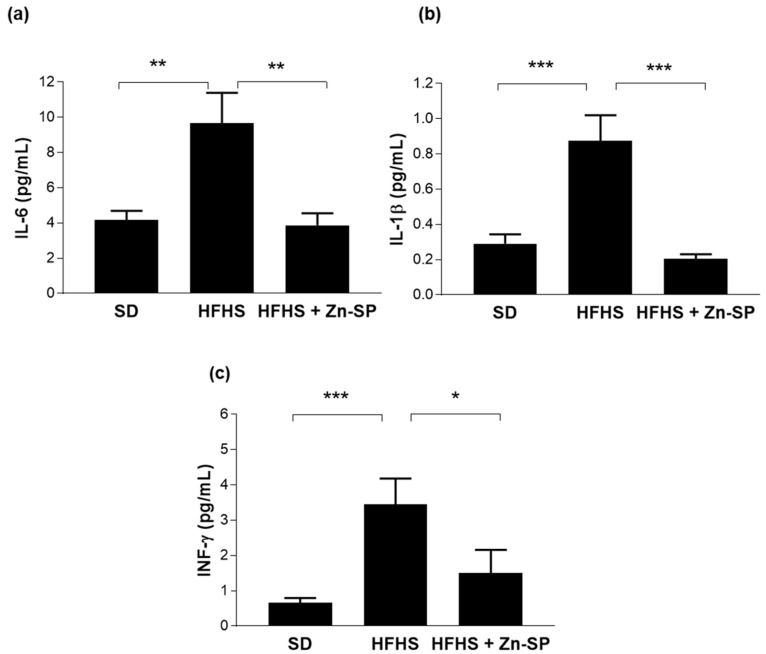
Markers of systemic inflammation. Plasmatic levels of IL-6 (**a**), IL-1β (**b**), and INFγ (**c**). Data are expressed as mean ± SEM of 5–10 mice per group. Statistical significance: * *p* < 0.05; ** *p* < 0.01; *** *p* < 0.001. SD: standard diet; HFHS: high-fat high-sugar diet; HFHS + Zn-SP: HFHS + zinc-enriched spirulina; IL-6: interleukin 6; IL-1β: interleukin 1beta; INF-γ: interferon gamma.

As shown in Figure 5, chronic exposure to HFHS diet resulted in a robust increase in plasma concentrations of pro-inflammatory cytokines, specifically IL-6, IFN-γ, and IL-1β. Intriguingly, administration of Zn-SP reported systemic cytokine concentration back to values similar to those detected in the plasma of SD mice.

**Figure 6:**
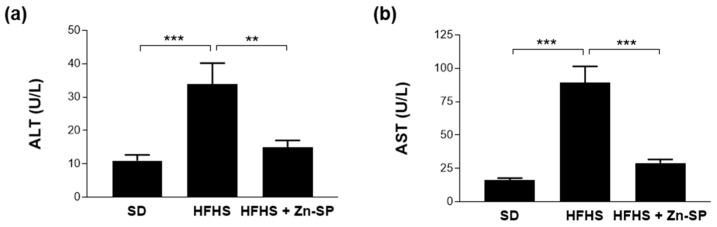
Markers of liver injury. Plasma levels of alanine transaminase (ALT) (**a**) and aspartate transaminase (AST) (**b**). All the data are expressed as mean ± SEM. Statistical significance: ** *p* < 0.01; *** *p* < 0.001. SD: standard diet; HFHS: high-fat high-sugar diet; HFHS + Zn-SP: HFHS + zinc-enriched spirulina; ALT: alanine aminotransferase; AST: aspartate aminotransferase.

Similarly, the diet-induced increase in blood concentrations of ALT and AST, well-known markers of hepatic injury (Figure 6), was significantly counteracted by the administration of Zn-SP.

### 3.6. Effects of Dietary Manipulation and Zn-SP Supplementation on Intestinal Microbiota

**Figure 7:**
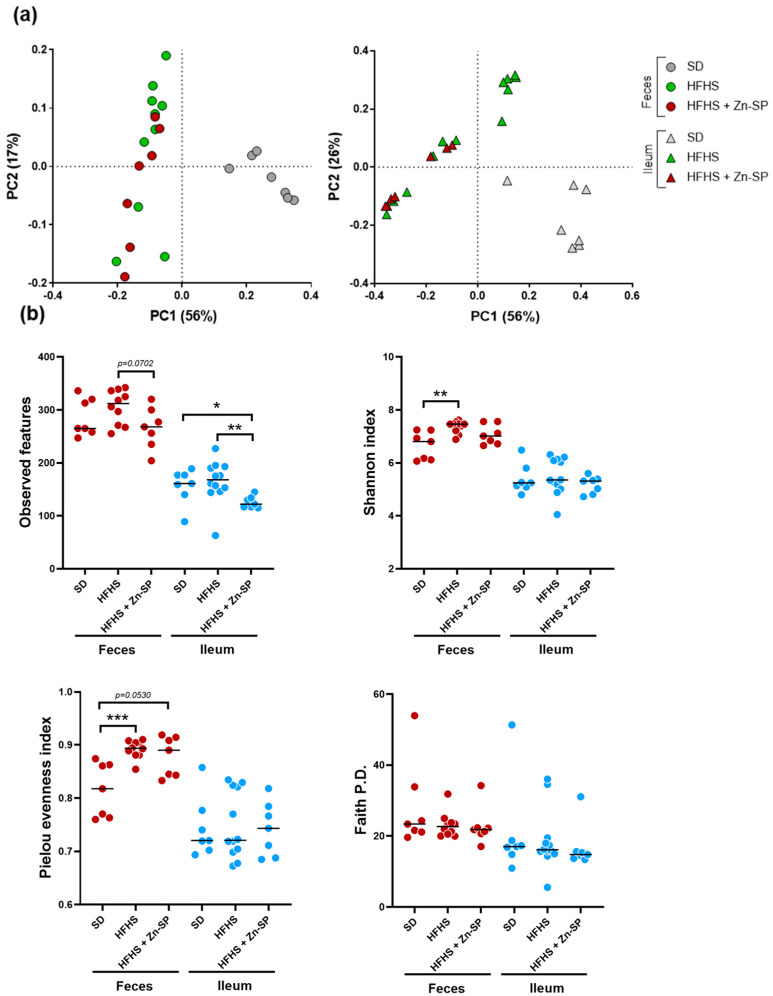
Diversity analysis of metataxomonic data. Metataxomonic data were obtained by 16S rRNA gene profiling carried out on fecal and ileum samples from mice fed a standard diet (SD), a high-fat and high-sugar diet (HFHS), and from mice that received a high-fat and high-sugar diet and lyophilized zinc-enriched spirulina (HFHS + Zn-SP). (**a**) Inter-sample (β-) diversity as determined through weighted UniFrac distance metric. (**b**) Intra-sample (α-) diversity assessed with four different indexes. Statistics is according to Mann–Whitney test; *, *p* < 0.05; **, *p* < 0.01; ***, *p* < 0.001.

The dietary intervention influenced the gut microbiota composition of mice in both feces and ileum, as evidenced by the β-diversity analysis based on weighted UniFrac distance metric. In fact, the taxonomic community structure of samples from SD mice clustered separately from HFHS mice (Figure 7a). In addition, α-diversity analysis revealed an increase in the evenness of the fecal bacterial abundances of HFHS mice, as evidenced by the significantly higher Shannon and Pielou indexes (Figure 7b). The administration of Zn-SP to HFHS mice reduced the evenness to levels not dissimilar to that of SD mice, and also determined a reduced richness in the ileum, according to a significantly lower observed features index (Figure 7b). In contrast, weighted UniFrac did not distinguish between the HFHS mice that received or did not receive the Zn-SP (Figure 7a).

Subsequently, we carried out a statistical analysis on CLR-transformed abundances to identify the bacterial taxa significantly different among the three groups of mice in feces and ileum. Notably, only 10 taxa were significantly differently represented in the feces between SD and HFHS mice, whereas HFHS + Zn-SP mice were characterized by 61 and 54 significantly different bacteria taxa compared to SD and HFHS mice, respectively (Supplementary Figure S1). In particular, HFHS + Zn-SP mice were characterized by the overrepresentation of the genera *Allobaculum* (family Erysipelotrichaceae) and *Adlercreutzia* (family Coriobacteriaceae).

In the ileum, SD mice had 36 and 29 differently represented taxa compared to HFHS and HFHS + Zn-SP mice, respectively, whereas 40 taxa were significantly different between HFHS and HFHS + Zn-SP mice (Supplementary Figure S2). The most evident difference in the ileal microbiota of HFHS + Zn-SP compared to HFHS mice was the increase in the family Rikenellaceae (phylum Bacteroidetes) and the reduced presence of members of the phylum Actinobacteria, the families Veillonellaceae (phylum Firmicutes) and Prevotellaceae (phylum Bacteroidetes), and the genera Prevotella and Fusobacterium (Supplementary Figure S2). A significant difference was also found between the HFHS and the HFHS + Zn-SP groups of mice for the Rikenellaceae/Veillonellaceae ratio (*p* = 0.0004; Supplementary Figure S3).

**Figure 8:**
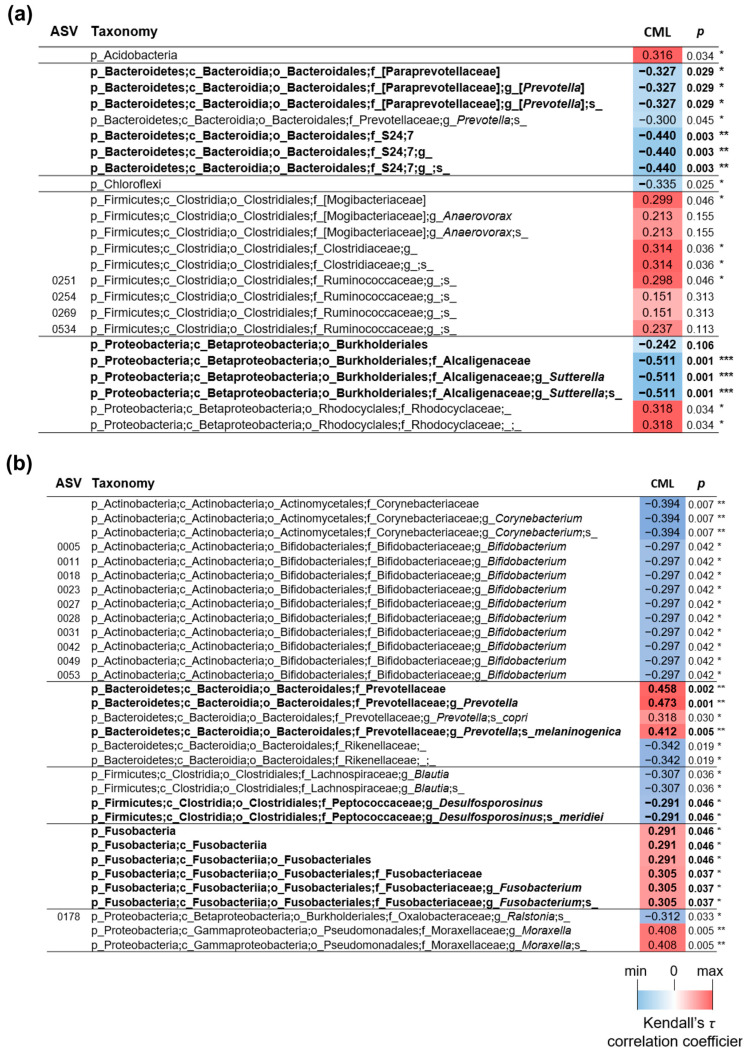
Correlation analysis between bacterial taxa and CML. The correlation analysis was performed between the CLR-transformed abundance of bacterial taxa in feces (**a**) and ileum (**b**) and the liver levels of Nε-carboxymethyl-lysine (CML) evaluated by LC-MS. Bacterial taxa that resulted differently represented between HFHS and HFHS + Zn-SP mice are indicated in bold. The taxonomic lineage of each taxon is as follows: p, phylum; c, class; o, order; f, family; g, genus; s, species. SD: standard diet; HFHS: high-fat high-sugar diet; HFHS + Zn-SP: HFHS + zinc-enriched spirulina. The blue-red heatmap refers to the Kendall’s τ rank correlation coefficient. The *p* value of each corresponding correlation coefficient is shown on the right; *, *p* < 0.05; **, *p* < 0.01; ***, *p* < 0.001.

Notably, several bacterial taxa that were significantly different between HFHS + Zn-SP compared to HFHS were also found to be significantly correlated with the liver levels of CML (Figure 8). In specific, in the fecal samples, the levels of the genus [*Prevotella*], the S24–7 family, and the genus *Sutterella* were all overrepresented in HFHS + Zn-SP compared to HFHS mice and were inversely correlated with CML liver levels (Figure 8a). Also, the species Desulfosporosinus meridei in the ileum was more represented in HFHS + Zn-SP mice and correlated inversely with CML levels (Figure 8b). In addition, in the ileum, the genus *Prevotella* (in particular, the species *P. melanogenica*) and the genus *Fusobacterium* were reduced in HFHS + Zn-SP mice and correlated positively with the liver levels of CML (Figure 8b).

## 4. Discussion

The significant changes that have occurred in the nutritional habits of modern countries in recent decades have worsened human health, increasing the incidence of chronic metabolic diseases. On the other hand, an increasing number of people turn to dietary supplementation to ameliorate their pathological conditions. Indeed, an incredible number of molecules of natural origin are provided with proven antioxidant and anti-inflammatory properties. In this perspective, nutrition research points to the characterization of foods or organic compounds that are particularly rich in bioactive molecules in high concentrations. Spirulina is a major potential dietary supplement with antioxidants and anti-inflammatory properties, thanks to its richness in many different bioactive ingredients [39,40].

In the present study, we confirmed and further extended these beneficial properties of spirulina platensis, showing a robust decrease in blood cytokines concentrations following Zn-SP administration to HFHS-fed mice. The well-known cross-talk mechanism linking the chronic low-grade inflammatory response evoked by dietary impairment, named metaflammation, to the development of insulin resistance is here further documented by findings showing that the diet-induced increase in systemic levels of cytokines was associated with significant impairment in fasting glucose concentrations and insulin sensitivity. Most notably, we documented a close parallelism between reduction in cytokines and improvement in glucose tolerance in HFHS-fed mice chronically exposed to Zn-SP. We also documented a significant improvement in HFHS-induced liver injury following Zn-SP administration, as documented by reduced systemic concentration of ALT and AST, thus suggesting the liver as one of the main organs that can benefit from this specific supplementation.

Although anti-obesity effects have been reported for spirulina in a few preclinical and clinical studies [41,42], here, we did not detect any impact of Zn-SP supplementation on body weight gain, thus highlighting how slight differences in the experimental models as well as in the kinetics of administration, doses or composition of the phytocomplex may affect spirulina’s ability to shape the metabolic profile.

Spirulina phytocomplex includes several antioxidant molecules such as phycocyanins, carotenoids, tocopherols, and phenols, and its efficacy in counteracting oxidative stress has been largely demonstrated in animal models and in human trials, showing that, thanks to its phytocomplex, spirulina can stimulate the transcription of antioxidant enzymes, such as catalase, superoxide dismutase, and glutathione peroxidase, and restore the amount of the reduced form of glutathione with respect to the oxidized [43]. Here, we report, for the first time, that the antioxidant effect of Zn-SP may be mediated by the enhancement of the transcriptional activity of Nrf2 in the liver. Nrf2 is responsible for the transcription of several antioxidant enzymes, as well as of enzymes for GSH recycling [44]. When we measured the GSSG/GSH ratio in the liver, we documented a significant restoration of GSH availability, thus confirming the antioxidant properties of the spirulina phytocomplex, which contribute, along with its anti-inflammatory properties, to protection against diet-induced metabolic derangements. It must be acknowledged that the spirulina we tested has been enriched in zinc which may further strengthen its beneficial effects. In fact, zinc acts on oxidative stress, being both an essential player in the process of antioxidant enzymes synthesis and an enzymatic catalyzer [45]. There is evidence that imbalances in zinc homeostasis are frequent in metabolic disorders [46,47] and blood zinc concentrations were found to be significantly decreased in obese patients [46,48]. Zinc supplementation in experimental diabetes has shown positive results improving glucose homeostasis [45]. However, there are still contrasting data about the clinical efficacy of zinc supplementation, with human trials on obese and diabetic patients reporting either positive outcomes or a lack of efficacy [49,50,51,52]. In mice fed the HFHS diet supplemented with Zn-SP, we observed a significant increase in plasma insulin levels, which may account for the enhanced peripheral glucose uptake. This effect could be due to either selective spirulina components, which have been recently shown to promote incretin-dependent insulin release by inhibiting the enzyme in charge for incretin metabolism, the dipeptidyl peptidase-4 (DPP-4) [53], and/or zinc, which has been proved to reduce hepatic insulin clearance, thus evoking apparently higher levels of insulin in peripheral blood [54]. 

Similarly, synergist effects between components of spirulina phytocomplex and zinc could contribute to the antioxidant effects here recorded. Indeed, in addition to its action as a cofactor of Cu/Zn-SOD and other antioxidant enzymes [47], zinc is reported to up-regulate Nrf2, thus increasing GSH synthesis and the transcription of the other target genes for antioxidant and detoxifying enzymes, including Glo-1 [55,56,57,58], which is the main enzyme responsible for the detoxification of the AGE precursors glyoxal and methylglyoxal, using GSH as cofactor. However, the lack of direct comparisons between mice fed with diets enriched only in zinc or spirulina does not allow us to draw definite conclusions about the added value of zinc supplementation in the formulation here tested.

Most notably, we documented for the first time that these beneficial antioxidant and anti-inflammatory effects of Zn-SP supplementation are due, at least in part, to its anti-glycation properties. The levels of two main AGE derivatives, CML and CEL, in the liver of HFHS + Zn-SP mice were similar to values recorded in control mice and significantly lower than that observed in mice fed the HFHS diet only. 

AGEs can exert their detrimental effect in tissues through direct glycation of proteins, altering their degradation or their function, or through binding to specific receptors, among which RAGE is the predominant AGE receptor [6]. Actually, the lower amount of AGEs in the liver of HFHS mice supplemented with Zn-SP was associated with severe inhibition of diet-induced over-expression of RAGE. RAGE is a receptor that is able to intercept different kinds of damage-associated molecular patterns, including AGEs [59,60]. In response to AGE binding, RAGE activates a wide range of signals leading to activation of pro-inflammatory transcription factors, including NFkB. NFkB in turn induces the transcription of proinflammatory cytokines and primes the assembling and activation of the NLRP3 inflammasome, one of the most characteristic pro-inflammatory molecular platforms involved in metaflammation onset [61,62,63]. Interestingly, the effective prevention of RAGE upregulation by Zn-SP was paralleled by robust reduction in both nuclear translocation of the NFκB-p65 and expression of NLRP3 inflammasome complex in the liver as well as by significant reduction in the systemic concentrations of NFκB- and NLRP3-dependent cytokines, IL-6/IFN-γ and IL-1β, respectively. Further insights are needed to elucidate the molecular mechanisms underlying Zn-SP ability to counteract AGE accumulation, most probably due to anti-glycating properties of selective components of the spirulina phytocomplex, such as phycocyanins, carotenoids, and polyphenols, which have already been documented to directly quench AGE dicarbonyl precursors [64,65,66,67]

The renowned property of spirulina to reshape gut microbiota, thanks to its content in non-digestible polysaccharides, may represent another relevant factor contributing to the beneficial effects of Zn-SP against AGE overproduction. Here, we confirmed the modulation of microbiota composition by Zn-SP towards increased microbial population abundance and restored Bacteroidetes/Firmicutes ratio, which has been previously demonstrated to ameliorate the glycative stress with reduced AGE levels and systemic inflammation [68]. Interestingly, as we and others have convincingly documented [11,69], the relationship between AGEs and gut microbiota can be bidirectional. In fact, the simple enrichment of the normocaloric SD with one specific class of AGEs can trigger a reshaping of the microbiota that is normally observed in the HFHS diet. A supposed explanation of this causal relationship is the potential selection of microbial species expressing methylglyoxal catabolic/synthesizing enzymes contributing to increased synthesis and systemic accumulation of AGEs [70,71,72]. In our study, we documented significant correlations between several bacterial taxa in both fecal and ileum samples and the level of the most representative AGE, CML, in the liver, thus confirming the cross-talk between altered microbial community profiles and AGEs and, most notably, strengthening the hypothesis that the beneficial outcomes of Zn-SP supplementation here recorded may be mediated, at least in part, by the reshaping of gut microbiota and the following impact on AGE accumulation.

## 5. Conclusions

Overall, our results could pave the way for the use of Zn-SP in preventive and/or adjuvant treatments for diet-induced metabolic derangements, showing that the antioxidant and anti-inflammatory effects of the proposed supplementation are related to the impairment of relevant signaling cascades modulating the expression of antioxidant enzymes and inflammatory mediators. Moreover, we report here for the first time that these effects are due, at least in part, to the Zn-SP ability to affect diet-induced AGE accumulation. Additional studies are needed to better elucidate the relative contribution of spirulina components and the zinc enrichment to the recorded protection in this specific pathological context.

## Figures and Tables

**Table 1:** Zinc-enriched *Arthrospira platensis* (spirulina) nutritional content.

Calories (Kcal/100 g) (Kj/100 g)	348 1480
Total carbohydrate (g/100 g) Dietary fiber Sugar	24.2 <1.0 <1.0
Total fat (g/100 g) Saturated fat	1.22 0.56
Protein (g/100 g)	60.05
Salt (g/100 g)	2.09
Iron (Fe) (mg/100 g)	27
Potassium (K) (mg/100 g)	1640
Magnesium (Mg) (mg/100 g)	304
Phosphorus (P) (mg/100 g)	830
Zinc (Zn) (mg/100 g)	200

**Table 2:** Physiological parameters. Data are expressed as mean ± SEM of 10 mice per group. Statistical analysis was performed using one-way ANOVA, followed by Bonferroni’s post hoc test, * *p* < 0.05. SD: standard diet; HFHS: high-fat high-sugar diet; HFHS + Zn-SP: HFHS + zinc-enriched spirulina.

	SD	HFHS	HFHS + Zn-SP
Diet energy supply (kcal/g)	3.85	5.56	5.56
Body weight (g)	28.37 ± 0.18	40.46 ± 0.76 *	40.86 ± 0.25 *
Food intake (g/day/mouse)	2.89 ± 0.05	3.10 ± 0.09	3.18 ± 0.15
Total calorie intake (kcal/day/mouse)	11.12 ± 0.20	17.26 ± 0.49 *	17.70 ± 0.85 *

## Data Availability

The data presented in this study are available on request from the corresponding author.

## Supplementary Materials

The following supporting information can be downloaded at: https://www.mdpi.com/article/10.3390/nu16040552/s1, Supplementary Table S1: List of primary antibodies; Supplementary Figure S1: Differences in bacteria taxa among experimental groups in feces; Supplementary Figure S2: Differences in bacteria taxa among experimental groups in ileum; Supplementary Figure S3: Rikenellaceae and Veilonellaceae abundances in the ileum of HFHS and HFHS + Zn-SP mice.
