# Supplementary material for: Anti-Glycation Properties of Zinc-Enriched Arthrospira platensis (Spirulina) Contribute to Prevention of Metaflammation in a Diet-Induced Obese Mouse Model
Source: Nutrients. 2024 Feb 17;16(4):552. doi: 10.3390/nu16040552 (PMC10892558; doi:10.3390/nu16040552)

Supplementary Table S1: List of primary antibodies used for Western blot technique. Primary antibodies were purchased from Abnova Corporation (Walnut, CA), Cosmo Bio (Carlsbad, CA), Santa Cruz Biotechnology, Inc. (Dallas,TX) , GeneTex, Inc. (Irvine, CA) Cell Signaling Technology, Inc. (Denver, MA), Adipogen Corporation(San Diego, CA).

**Code**	**Antibody**
PAB1293	anti-CML
KAL-KH025	anti-CEL
sc-365154	anti-RAGE
sc-13032	anti-Nrf2
Gtx15747	anti-Glo-1
8242S	anti-NFκB p65
AG-20B-0014-C100	anti-NLRP3
93709	anti-Gasdermin
5174S	anti-GAPDH
4499S	anti-Histone H3

Supplementary Figure S1: Bacterial taxa that resulted differently represented among experimental groups in feces. ASV, Amplicon Sequence Variants. P, statistical significance according to Mann-Whitney test performed on centered log-ratio (CLR)-transformed abundances.

[SD] vs [HFHS]				
**ASV**	**Taxonomy**	***P***	**SD**	**HFHS**
	p_Acidobacteria	0.0432	0.00	0.32
	p_Actinobacteria;c_Actinobacteria;o_Bifidobacteriales;f_Bifidobacteriaceae;g_Bifidobacterium;s_	0.0395	0.34	0.00
	p_Firmicutes;c_Bacilli;o_Bacillales;f_Staphylococcaceae;g_Staphylococcus	0.0393	0.00	0.74
	p_Firmicutes;c_Bacilli;o_Bacillales;f_Staphylococcaceae;g_Staphylococcus;s_sciuri	0.0432	0.00	0.68
	p_Firmicutes;c_Bacilli;o_Lactobacillales;f_Enterococcaceae	0.0395	0.00	0.18
	p_Firmicutes;c_Bacilli;o_Lactobacillales;f_Enterococcaceae;_	0.0395	0.00	0.18
	p_Firmicutes;c_Bacilli;o_Lactobacillales;f_Enterococcaceae;_;_	0.0395	0.00	0.18
	p_Firmicutes;c_Clostridia;o_Clostridiales;f_Lachnospiraceae;g_;Ruminococcus;;s_	0.0395	0.00	0.64
	p_Proteobacteria;c_Deltaproteobacteria;o_Desulfovibrionales;f_Desulfovibrionaceae;g_	0.0110	1.32	0.00
	p_Proteobacteria;c_Deltaproteobacteria;o_Desulfovibrionales;f_Desulfovibrionaceae;g_;s_	0.0110	1.32	0.00

[SD] vs [HFHS+Zn-SP]				
**ASV**	**Taxonomy**	***P***	**SD**	**HFHS+ Zn-SP**
	p_Actinobacteria	0.0070	6.24	4.77
_0011	p_Actinobacteria;c_Actinobacteria;o_Bifidobacteriales;f_Bifidobacteriaceae;g_Bifidobacterium	0.0236	2.42	0.00
_0028	p_Actinobacteria;c_Actinobacteria;o_Bifidobacteriales;f_Bifidobacteriaceae;g_Bifidobacterium	0.0307	0.73	0.00
_0053	p_Actinobacteria;c_Actinobacteria;o_Bifidobacteriales;f_Bifidobacteriaceae;g_Bifidobacterium	0.0307	0.63	0.00
	p_Actinobacteria;c_Actinobacteria;o_Bifidobacteriales;f_Bifidobacteriaceae;g_Bifidobacterium;s_	0.0307	0.34	0.00
	p_Actinobacteria;c_Coriobacteriia;o_Coriobacteriales;f_Coriobacteriaceae;g_Adlercreutzia	0.0262	2.33	4.35
	p_Actinobacteria;c_Coriobacteriia;o_Coriobacteriales;f_Coriobacteriaceae;g_Adlercreutzia;s_	0.0262	2.33	4.35
_0090	p_Actinobacteria;c_Coriobacteriia;o_Coriobacteriales;f_Coriobacteriaceae;g_Adlercreutzia;s_	0.0307	0.00	1.22
_0135	p_Actinobacteria;c_Coriobacteriia;o_Coriobacteriales;f_Coriobacteriaceae;g_Adlercreutzia;s_	0.0307	0.00	0.64
	p_Actinobacteria;c_Coriobacteriia;o_Coriobacteriales;f_Coriobacteriaceae;g_Collinsella	0.0291	0.57	1.23
	p_Actinobacteria;c_Coriobacteriia;o_Coriobacteriales;f_Coriobacteriaceae;g_Collinsella;s_aerofaciens	0.0114	0.00	0.79
	p_Bacteroidetes;c_Bacteroidia;o_Bacteroidales;f_[Odoribacteraceae]	0.0338	0.00	0.78
	p_Bacteroidetes;c_Bacteroidia;o_Bacteroidales;f_[Odoribacteraceae];g_Odoribacter	0.0338	0.00	0.78
	p_Bacteroidetes;c_Bacteroidia;o_Bacteroidales;f_[Odoribacteraceae];g_Odoribacter;s_	0.0338	0.00	0.78
	p_Bacteroidetes;c_Bacteroidia;o_Bacteroidales;f_Bacteroidaceae	0.0111	3.23	2.66
	p_Bacteroidetes;c_Bacteroidia;o_Bacteroidales;f_Bacteroidaceae;g_Bacteroides	0.0111	3.23	2.66
	p_Bacteroidetes;c_Bacteroidia;o_Bacteroidales;f_Bacteroidaceae;g_Bacteroides;s_	0.0379	2.71	2.17
	p_Bacteroidetes;c_Bacteroidia;o_Bacteroidales;f_Prevotellaceae;g_Prevotella;s_melaninogenica	0.0209	0.98	1.42
	p_Bacteroidetes;c_Bacteroidia;o_Bacteroidales;f_Rikenellaceae	0.0070	3.17	2.60
	p_Bacteroidetes;c_Bacteroidia;o_Bacteroidales;f_Rikenellaceae;g_	0.0070	3.07	2.51
	p_Bacteroidetes;c_Bacteroidia;o_Bacteroidales;f_Rikenellaceae;g_;s_	0.0070	3.07	2.51
	p_Firmicutes	0.0070	6.15	7.13
	p_Firmicutes;c_Bacilli	0.0070	3.73	4.20
	p_Firmicutes;c_Bacilli;o_Lactobacillales	0.0070	3.72	4.01
_0139	p_Firmicutes;c_Bacilli;o_Lactobacillales;f_Lactobacillaceae;g_Lactobacillus;s_	0.0307	0.96	0.00
	p_Firmicutes;c_Clostridia	0.0262	5.78	5.02
	p_Firmicutes;c_Clostridia;o_Clostridiales	0.0262	5.78	5.02
	p_Firmicutes;c_Clostridia;o_Clostridiales;f_	0.0111	4.84	4.30
	p_Firmicutes;c_Clostridia;o_Clostridiales;f_;g_	0.0111	4.84	4.30
	p_Firmicutes;c_Clostridia;o_Clostridiales;f_;g_;s_	0.0111	4.84	4.30
	p_Firmicutes;c_Clostridia;o_Clostridiales;f_;Mogibacteriaceae;;g_Anaerovorax	0.0307	0.23	0.00
	p_Firmicutes;c_Clostridia;o_Clostridiales;f_;Mogibacteriaceae;;g_Anaerovorax;s_	0.0307	0.23	0.00
	p_Firmicutes;c_Clostridia;o_Clostridiales;f_Dehalobacteriaceae	0.0187	1.26	0.00
	p_Firmicutes;c_Clostridia;o_Clostridiales;f_Dehalobacteriaceae;g_Dehalobacterium	0.0187	1.26	0.00
	p_Firmicutes;c_Clostridia;o_Clostridiales;f_Dehalobacteriaceae;g_Dehalobacterium;s_	0.0187	1.26	0.00
	p_Firmicutes;c_Clostridia;o_Clostridiales;f_Lachnospiraceae	0.0175	3.87	2.95
	p_Firmicutes;c_Clostridia;o_Clostridiales;f_Lachnospiraceae;g_	0.0175	3.00	1.52
	p_Firmicutes;c_Clostridia;o_Clostridiales;f_Lachnospiraceae;g_;s_	0.0175	3.00	1.52
	p_Firmicutes;c_Clostridia;o_Clostridiales;f_Lachnospiraceae;g_Coprococcus	0.0476	1.11	0.00
	p_Firmicutes;c_Clostridia;o_Clostridiales;f_Lachnospiraceae;g_Coprococcus;s_	0.0476	1.11	0.00
	p_Firmicutes;c_Clostridia;o_Clostridiales;f_Ruminococcaceae	0.0070	4.84	3.62
	p_Firmicutes;c_Clostridia;o_Clostridiales;f_Ruminococcaceae;g_	0.0041	4.02	2.88
	p_Firmicutes;c_Clostridia;o_Clostridiales;f_Ruminococcaceae;g_;s_	0.0041	4.02	2.88
	p_Firmicutes;c_Clostridia;o_Clostridiales;f_Ruminococcaceae;g_Ruminococcus	0.0452	2.39	0.81
	p_Firmicutes;c_Erysipelotrichi	0.0041	4.30	6.95
	p_Firmicutes;c_Erysipelotrichi;o_Erysipelotrichales	0.0041	4.30	6.95
	p_Firmicutes;c_Erysipelotrichi;o_Erysipelotrichales;f_Erysipelotrichaceae	0.0041	4.30	6.95
	p_Firmicutes;c_Erysipelotrichi;o_Erysipelotrichales;f_Erysipelotrichaceae;g_Allobaculum	0.0041	4.24	6.95
	p_Firmicutes;c_Erysipelotrichi;o_Erysipelotrichales;f_Erysipelotrichaceae;g_Allobaculum;s_	0.0041	4.24	6.95
	p_Proteobacteria;c_Alphaproteobacteria;o_RF32	0.0175	1.02	1.30
	p_Proteobacteria;c_Alphaproteobacteria;o_RF32;f_	0.0175	1.02	1.30
	p_Proteobacteria;c_Alphaproteobacteria;o_RF32;f_;g_	0.0175	1.02	1.30
	p_Proteobacteria;c_Alphaproteobacteria;o_RF32;f_;g_;s_	0.0175	1.02	1.30
	p_Proteobacteria;c_Betaproteobacteria	0.0379	2.98	3.41
	p_Proteobacteria;c_Betaproteobacteria;o_Burkholderiales	0.0070	2.18	2.74
	p_Tenericutes	0.0204	0.67	0.00
	p_Tenericutes;c_Mollicutes	0.0204	0.67	0.00
	p_Tenericutes;c_Mollicutes;o_Anaeroplasmatales	0.0307	0.67	0.00
	p_Tenericutes;c_Mollicutes;o_Anaeroplasmatales;f_Anaeroplasmataceae	0.0307	0.67	0.00
	p_Tenericutes;c_Mollicutes;o_Anaeroplasmatales;f_Anaeroplasmataceae;g_Anaeroplasma	0.0307	0.67	0.00
	p_Tenericutes;c_Mollicutes;o_Anaeroplasmatales;f_Anaeroplasmataceae;g_Anaeroplasma;s_	0.0307	0.67	0.00

[HFHS] vs [HFHS+Zn-SP]				
**ASV**	**Taxonomy**	***P***	**HFHS**	**HFHS+ Zn-SP**
_0031	p_Actinobacteria;c_Actinobacteria;o_Bifidobacteriales;f_Bifidobacteriaceae;g_Bifidobacterium	0.0432	1.86	0.00
	p_Actinobacteria;c_Coriobacteriia;o_Coriobacteriales;f_Coriobacteriaceae;g_Adlercreutzia	0.0001	2.38	4.35
	p_Actinobacteria;c_Coriobacteriia;o_Coriobacteriales;f_Coriobacteriaceae;g_Adlercreutzia;s_	0.0001	2.38	4.35
_0090	p_Actinobacteria;c_Coriobacteriia;o_Coriobacteriales;f_Coriobacteriaceae;g_Adlercreutzia;s_	0.0293	0.00	1.22
	p_Actinobacteria;c_Coriobacteriia;o_Coriobacteriales;f_Coriobacteriaceae;g_Collinsella;s_aerofaciens	0.0192	0.00	0.79
	p_Bacteroidetes;c_Bacteroidia;o_Bacteroidales;f_[Odoribacteraceae]	0.0140	0.00	0.78
	p_Bacteroidetes;c_Bacteroidia;o_Bacteroidales;f_[Odoribacteraceae];g_Odoribacter	0.0140	0.00	0.78
	p_Bacteroidetes;c_Bacteroidia;o_Bacteroidales;f_[Odoribacteraceae];g_Odoribacter;s_	0.0140	0.00	0.78
	p_Bacteroidetes;c_Bacteroidia;o_Bacteroidales;f_[Paraprevotellaceae]	0.0072	0.99	1.70
	p_Bacteroidetes;c_Bacteroidia;o_Bacteroidales;f_[Paraprevotellaceae];g_[Prevotella]	0.0072	0.99	1.70
	p_Bacteroidetes;c_Bacteroidia;o_Bacteroidales;f_[Paraprevotellaceae];g_[Prevotella];s_	0.0072	0.99	1.70
	p_Bacteroidetes;c_Bacteroidia;o_Bacteroidales;f_Porphyromonadaceae;g_Parabacteroides	0.0336	1.78	0.56
	p_Bacteroidetes;c_Bacteroidia;o_Bacteroidales;f_Porphyromonadaceae;g_Parabacteroides;s_	0.0262	1.78	0.56
	p_Bacteroidetes;c_Bacteroidia;o_Bacteroidales;f_Prevotellaceae	0.0185	1.33	1.83
	p_Bacteroidetes;c_Bacteroidia;o_Bacteroidales;f_Prevotellaceae;g_Prevotella	0.0185	1.33	1.83
	p_Bacteroidetes;c_Bacteroidia;o_Bacteroidales;f_Rikenellaceae;_	0.0106	0.00	0.66
	p_Bacteroidetes;c_Bacteroidia;o_Bacteroidales;f_Rikenellaceae;_;_	0.0106	0.00	0.66
	p_Bacteroidetes;c_Bacteroidia;o_Bacteroidales;f_S24-7	0.0185	4.16	4.95
	p_Bacteroidetes;c_Bacteroidia;o_Bacteroidales;f_S24-7;g_	0.0185	4.16	4.95
	p_Bacteroidetes;c_Bacteroidia;o_Bacteroidales;f_S24-7;g_;s_	0.0185	4.16	4.95
_0100	p_Bacteroidetes;c_Bacteroidia;o_Bacteroidales;f_S24-7;g_;s_	0.0106	0.00	1.24
_0151	p_Bacteroidetes;c_Bacteroidia;o_Bacteroidales;f_S24-7;g_;s_	0.0334	0.00	0.00
_0234	p_Bacteroidetes;c_Bacteroidia;o_Bacteroidales;f_S24-7;g_;s_	0.0334	0.00	0.00
	p_Firmicutes	0.0020	6.10	7.13
	p_Firmicutes;c_Bacilli	0.0431	3.87	4.20
	p_Firmicutes;c_Bacilli;o_Lactobacillales;f_Lactobacillaceae	0.0330	3.17	3.52
	p_Firmicutes;c_Bacilli;o_Lactobacillales;f_Lactobacillaceae;g_Lactobacillus	0.0330	3.17	3.52
	p_Firmicutes;c_Clostridia	0.0431	5.69	5.02
	p_Firmicutes;c_Clostridia;o_Clostridiales	0.0431	5.69	5.02
	p_Firmicutes;c_Clostridia;o_Clostridiales;f_Clostridiaceae	0.0146	2.02	0.85
	p_Firmicutes;c_Clostridia;o_Clostridiales;f_Lachnospiraceae;g_	0.0250	2.76	1.52
	p_Firmicutes;c_Clostridia;o_Clostridiales;f_Lachnospiraceae;g_;s_	0.0250	2.76	1.52
	p_Firmicutes;c_Clostridia;o_Clostridiales;f_Lachnospiraceae;g_Coprococcus	0.0432	2.05	0.00
	p_Firmicutes;c_Clostridia;o_Clostridiales;f_Lachnospiraceae;g_Coprococcus;s_	0.0432	2.05	0.00
	p_Firmicutes;c_Clostridia;o_Clostridiales;f_Ruminococcaceae;g_Ruminococcus	0.0071	2.37	0.81
	p_Firmicutes;c_Clostridia;o_Clostridiales;f_Ruminococcaceae;g_Ruminococcus;s_	0.0320	2.30	0.00
	p_Firmicutes;c_Erysipelotrichi	0.0012	4.28	6.95
	p_Firmicutes;c_Erysipelotrichi;o_Erysipelotrichales	0.0012	4.28	6.95
	p_Firmicutes;c_Erysipelotrichi;o_Erysipelotrichales;f_Erysipelotrichaceae	0.0012	4.28	6.95
	p_Firmicutes;c_Erysipelotrichi;o_Erysipelotrichales;f_Erysipelotrichaceae;g_Allobaculum	0.0012	4.10	6.95
	p_Firmicutes;c_Erysipelotrichi;o_Erysipelotrichales;f_Erysipelotrichaceae;g_Allobaculum;s_	0.0012	4.10	6.95
	p_Proteobacteria;c_Betaproteobacteria	0.0431	3.03	3.41
	p_Proteobacteria;c_Betaproteobacteria;o_Burkholderiales	0.0031	2.15	2.74
	p_Proteobacteria;c_Betaproteobacteria;o_Burkholderiales;f_Alcaligenaceae	0.0063	0.00	0.82
	p_Proteobacteria;c_Betaproteobacteria;o_Burkholderiales;f_Alcaligenaceae;g_Sutterella	0.0063	0.00	0.82
	p_Proteobacteria;c_Betaproteobacteria;o_Burkholderiales;f_Alcaligenaceae;g_Sutterella;s_	0.0063	0.00	0.82
	p_Proteobacteria;c_Betaproteobacteria;o_Burkholderiales;f_Comamonadaceae	0.0330	1.00	1.29
	p_Proteobacteria;c_Betaproteobacteria;o_Burkholderiales;f_Comamonadaceae;g_	0.0280	0.98	1.27
	p_Proteobacteria;c_Betaproteobacteria;o_Burkholderiales;f_Comamonadaceae;g_;s_	0.0280	0.98	1.27
	p_Proteobacteria;c_Betaproteobacteria;o_Burkholderiales;f_Oxalobacteraceae	0.0453	1.80	2.27
	p_Proteobacteria;c_Betaproteobacteria;o_Burkholderiales;f_Oxalobacteraceae;g_Ralstonia	0.0449	1.80	2.27
	p_Proteobacteria;c_Betaproteobacteria;o_Burkholderiales;f_Oxalobacteraceae;g_Ralstonia;s_	0.0449	1.80	2.27
	p_Proteobacteria;c_Deltaproteobacteria;o_Desulfovibrionales;f_Desulfovibrionaceae;g_	0.0132	0.00	1.31
	p_Proteobacteria;c_Deltaproteobacteria;o_Desulfovibrionales;f_Desulfovibrionaceae;g_;s_	0.0132	0.00	1.31

0 max

Median of CLR-transformed abundaces

Supplementary Figure S2: Bacterial taxa that resulted differently represented among experimental groups in ileum. ASV, Amplicon Sequence Variants. P, statistical significance according to Mann-Whitney test performed on centered log-ratio (CLR)-transformed abundances.

[SD] vs [HFHS]

ASV	Taxonomy	*P*	SD	HFHS
_0020	p_Actinobacteria;c_Coriobacteriia;o_Coriobacteriales;f_Coriobacteriaceae;g_;s_	0.0292	1.60	0.00
_0058	p_Actinobacteria;c_Coriobacteriia;o_Coriobacteriales;f_Coriobacteriaceae;g_;s_	0.0292	1.01	0.00
_0072	p_Actinobacteria;c_Coriobacteriia;o_Coriobacteriales;f_Coriobacteriaceae;g_Adlercreutzia;s_	0.0048	1.43	0.00
_0098	p_Actinobacteria;c_Coriobacteriia;o_Coriobacteriales;f_Coriobacteriaceae;g_Adlercreutzia;s_	0.0105	1.76	0.00
_0135	p_Actinobacteria;c_Coriobacteriia;o_Coriobacteriales;f_Coriobacteriaceae;g_Adlercreutzia;s_	0.0358	0.62	0.00
	p_Bacteroidetes;c_Bacteroidia;o_Bacteroidales;f_Prevotellaceae;g_Prevotella;s_copri	0.0398	0.00	0.48
	p_Firmicutes;c_Bacilli;o_Gemellales	0.0106	0.00	1.79
	p_Firmicutes;c_Bacilli;o_Gemellales;f_Gemellaceae	0.0106	0.00	1.79
	p_Firmicutes;c_Bacilli;o_Gemellales;f_Gemellaceae;g_	0.0106	0.00	1.79
	p_Firmicutes;c_Bacilli;o_Gemellales;f_Gemellaceae;g_;s_	0.0106	0.00	1.79
_0110	p_Firmicutes;c_Bacilli;o_Lactobacillales;f_Lactobacillaceae;g_Lactobacillus;s_	0.0458	1.37	0.00
	p_Firmicutes;c_Clostridia;o_Clostridiales;f_Lachnospiraceae;g_[Ruminococcus];s_gnavus	0.0304	0.00	2.74
	p_Firmicutes;c_Clostridia;o_Clostridiales;f_Veillonellaceae	0.0283	2.25	3.61
	p_Firmicutes;c_Clostridia;o_Clostridiales;f_Veillonellaceae;g_Dialister	0.0216	0.00	1.37
	p_Firmicutes;c_Clostridia;o_Clostridiales;f_Veillonellaceae;g_Dialister;s_	0.0216	0.00	1.37
	p_Firmicutes;c_Clostridia;o_Clostridiales;f_Veillonellaceae;g_Megamonas	0.0252	0.00	2.33
	p_Firmicutes;c_Clostridia;o_Clostridiales;f_Veillonellaceae;g_Megamonas;s_	0.0252	0.00	2.33
	p_Firmicutes;c_Clostridia;o_Clostridiales;f_Veillonellaceae;g_Megasphaera	0.0028	0.00	1.79
	p_Firmicutes;c_Clostridia;o_Clostridiales;f_Veillonellaceae;g_Megasphaera;s_	0.0028	0.00	1.79
	p_Firmicutes;c_Clostridia;o_Clostridiales;f_Veillonellaceae;g_Phascolarctobacterium	0.0351	0.00	1.91
	p_Firmicutes;c_Clostridia;o_Clostridiales;f_Veillonellaceae;g_Phascolarctobacterium;s_	0.0351	0.00	1.91
_0001	p_Firmicutes;c_Erysipelotrichi;o_Erysipelotrichales;f_Erysipelotrichaceae;g_Allobaculum;s_	0.0140	3.68	0.00
_0004	p_Firmicutes;c_Erysipelotrichi;o_Erysipelotrichales;f_Erysipelotrichaceae;g_Allobaculum;s_	0.0179	3.56	0.00
_0008	p_Firmicutes;c_Erysipelotrichi;o_Erysipelotrichales;f_Erysipelotrichaceae;g_Allobaculum;s_	0.0109	3.39	0.00
_0009	p_Firmicutes;c_Erysipelotrichi;o_Erysipelotrichales;f_Erysipelotrichaceae;g_Allobaculum;s_	0.0109	3.42	0.00
_0024	p_Firmicutes;c_Erysipelotrichi;o_Erysipelotrichales;f_Erysipelotrichaceae;g_Allobaculum;s_	0.0227	2.84	0.00
_0030	p_Firmicutes;c_Erysipelotrichi;o_Erysipelotrichales;f_Erysipelotrichaceae;g_Allobaculum;s_	0.0084	2.83	0.00
	p_Proteobacteria;c_Deltaproteobacteria;o_Desulfovibrionales;f_Desulfovibrionaceae;g_	0.0358	1.11	0.00
	p_Proteobacteria;c_Deltaproteobacteria;o_Desulfovibrionales;f_Desulfovibrionaceae;g_;s_	0.0358	1.11	0.00
	p_Proteobacteria;c_Gammaproteobacteria	0.0052	2.37	2.88
	p_Proteobacteria;c_Gammaproteobacteria;o_Enterobacteriales	0.0437	0.00	2.06
	p_Proteobacteria;c_Gammaproteobacteria;o_Enterobacteriales;f_Enterobacteriaceae	0.0437	0.00	2.06
	p_Proteobacteria;c_Gammaproteobacteria;o_Enterobacteriales;f_Enterobacteriaceae;g_	0.0437	0.00	2.06
	p_Proteobacteria;c_Gammaproteobacteria;o_Enterobacteriales;f_Enterobacteriaceae;g_;s_	0.0437	0.00	2.06
	p_Proteobacteria;c_Gammaproteobacteria;o_Pseudomonadales;f_Moraxellaceae;g_Moraxella	0.0357	0.00	0.33
	p_Proteobacteria;c_Gammaproteobacteria;o_Pseudomonadales;f_Moraxellaceae;g_Moraxella;s_	0.0357	0.00	0.33

[SD] vs [HFHS+Zn-SP]

ASV	Taxonomy	*P*	SD	HFHS+Zn-SP
	p_Actinobacteria	0.0262	6.99	3.57
	p_Actinobacteria;c_Actinobacteria;o_Actinomycetales;f_Actinomycetaceae	0.0407	0.00	0.95
	p_Actinobacteria;c_Actinobacteria;o_Actinomycetales;f_Actinomycetaceae;g_Actinomyces	0.0407	0.00	0.95
	p_Actinobacteria;c_Actinobacteria;o_Actinomycetales;f_Actinomycetaceae;g_Actinomyces;s_	0.0407	0.00	0.95
	p_Actinobacteria;c_Coriobacteriia	0.0379	3.74	2.91
	p_Actinobacteria;c_Coriobacteriia;o_Coriobacteriales	0.0379	3.74	2.91
	p_Actinobacteria;c_Coriobacteriia;o_Coriobacteriales;f_Coriobacteriaceae	0.0379	3.74	2.91
	p_Actinobacteria;c_Coriobacteriia;o_Coriobacteriales;f_Coriobacteriaceae;g_	0.0474	3.04	1.71
	p_Actinobacteria;c_Coriobacteriia;o_Coriobacteriales;f_Coriobacteriaceae;g_;s_	0.0474	3.04	1.71
	p_Bacteroidetes;c_Bacteroidia;o_Bacteroidales;f_	0.0238	0.00	0.40
	p_Bacteroidetes;c_Bacteroidia;o_Bacteroidales;f_;g_	0.0238	0.00	0.40
	p_Bacteroidetes;c_Bacteroidia;o_Bacteroidales;f_;g_;s_	0.0238	0.00	0.40
	p_Bacteroidetes;c_Bacteroidia;o_Bacteroidales;f_Prevotellaceae;g_Prevotella;s_	0.0338	0.63	0.00
	p_Firmicutes;c_Clostridia	0.0379	5.01	5.81
	p_Firmicutes;c_Clostridia;o_Clostridiales	0.0379	5.01	5.81
	p_Firmicutes;c_Clostridia;o_Clostridiales;f_	0.0262	4.10	4.73
	p_Firmicutes;c_Clostridia;o_Clostridiales;f_;g_	0.0262	4.10	4.73
	p_Firmicutes;c_Clostridia;o_Clostridiales;f_;g_;s_	0.0262	4.10	4.73
	p_Firmicutes;c_Clostridia;o_Clostridiales;f_Clostridiaceae	0.0282	0.00	0.83
	p_Firmicutes;c_Clostridia;o_Clostridiales;f_Lachnospiraceae;g_Dorea	0.0199	0.00	1.56
	p_Firmicutes;c_Clostridia;o_Clostridiales;f_Lachnospiraceae;g_Dorea;s_	0.0199	0.00	1.56
	p_Firmicutes;c_Clostridia;o_Clostridiales;f_Ruminococcaceae	0.0379	3.61	4.82
	p_Firmicutes;c_Clostridia;o_Clostridiales;f_Ruminococcaceae;g_Oscillospira	0.0387	0.00	3.96
	p_Firmicutes;c_Clostridia;o_Clostridiales;f_Ruminococcaceae;g_Oscillospira;s_	0.0387	0.00	3.96
	p_Firmicutes;c_Clostridia;o_Clostridiales;f_Ruminococcaceae;g_Ruminococcus	0.0330	0.93	2.45
	p_Firmicutes;c_Clostridia;o_Clostridiales;f_Ruminococcaceae;g_Ruminococcus;s_	0.0263	0.00	2.45
	p_Firmicutes;c_Clostridia;o_Clostridiales;f_Veillonellaceae;g_Megasphaera	0.0407	0.00	0.66
	p_Firmicutes;c_Clostridia;o_Clostridiales;f_Veillonellaceae;g_Megasphaera;s_	0.0407	0.00	0.66
_0105	p_Proteobacteria;c_Betaproteobacteria;o_Burkholderiales;f_Oxalobacteraceae;g_Ralstonia;s_	0.0407	0.00	1.99

[HFHS] vs [HFHS+Zn-SP]				
**ASV**	**Taxonomy**	***P***	**HFHS**	**HFHS+Zn-SP**
	p_Actinobacteria	0.0072	6.36	3.57
_0072	p_Actinobacteria;c_Coriobacteriia;o_Coriobacteriales;f_Coriobacteriaceae;g_Adlercreutzia;s_	0.0292	0.00	0.81
	p_Bacteroidetes;c_Bacteroidia;o_Bacteroidales;f_	0.0135	0.00	0.40
	p_Bacteroidetes;c_Bacteroidia;o_Bacteroidales;f_;g_	0.0135	0.00	0.40
	p_Bacteroidetes;c_Bacteroidia;o_Bacteroidales;f_;g_;s_	0.0135	0.00	0.40
	p_Bacteroidetes;c_Bacteroidia;o_Bacteroidales;f_Bacteroidaceae;g_Bacteroides;s_fragilis	0.0375	1.53	0.06
	p_Bacteroidetes;c_Bacteroidia;o_Bacteroidales;f_Prevotellaceae	0.0037	2.03	1.34
	p_Bacteroidetes;c_Bacteroidia;o_Bacteroidales;f_Prevotellaceae;g_Prevotella	0.0026	2.03	1.31
	p_Bacteroidetes;c_Bacteroidia;o_Bacteroidales;f_Prevotellaceae;g_Prevotella;s_melaninogenica	0.0221	1.40	1.10
	p_Bacteroidetes;c_Bacteroidia;o_Bacteroidales;f_Rikenellaceae	0.0358	2.02	3.48
	p_Bacteroidetes;c_Bacteroidia;o_Bacteroidales;f_Rikenellaceae;g_	0.0358	2.02	3.48
	p_Bacteroidetes;c_Bacteroidia;o_Bacteroidales;f_Rikenellaceae;g_;s_	0.0358	2.02	3.48
_0100	p_Bacteroidetes;c_Bacteroidia;o_Bacteroidales;f_S24;7;g_;s_	0.0497	0.00	0.58
	p_Firmicutes;c_Bacilli;o_Bacillales	0.0449	1.62	1.17
	p_Firmicutes;c_Bacilli;o_Bacillales;f_Bacillaceae	0.0283	1.53	1.17
	p_Firmicutes;c_Bacilli;o_Bacillales;f_Bacillaceae;g_Bacillus	0.0283	1.53	1.17
	p_Firmicutes;c_Bacilli;o_Bacillales;f_Bacillaceae;g_Bacillus;s_	0.0311	1.53	1.17
	p_Firmicutes;c_Bacilli;o_Gemellales	0.0381	1.79	0.62
	p_Firmicutes;c_Bacilli;o_Gemellales;f_Gemellaceae	0.0381	1.79	0.62
	p_Firmicutes;c_Bacilli;o_Gemellales;f_Gemellaceae;g_	0.0381	1.79	0.62
	p_Firmicutes;c_Bacilli;o_Gemellales;f_Gemellaceae;g_;s_	0.0381	1.79	0.62
	p_Firmicutes;c_Clostridia;o_Clostridiales;f_Peptococcaceae;g_Desulfosporosinus	0.0497	0.00	0.51
	p_Firmicutes;c_Clostridia;o_Clostridiales;f_Peptococcaceae;g_Desulfosporosinus;s_meridiei	0.0497	0.00	0.51
	p_Firmicutes;c_Clostridia;o_Clostridiales;f_Veillonellaceae	0.0449	3.61	2.29
	p_Firmicutes;c_Clostridia;o_Clostridiales;f_Veillonellaceae;g_Dialister	0.0351	1.37	0.00
	p_Firmicutes;c_Clostridia;o_Clostridiales;f_Veillonellaceae;g_Dialister;s_	0.0351	1.37	0.00
	p_Fusobacteria	0.0283	2.07	1.49
	p_Fusobacteria;c_Fusobacteriia	0.0283	2.07	1.49
	p_Fusobacteria;c_Fusobacteriia;o_Fusobacteriales	0.0283	2.07	1.49
	p_Fusobacteria;c_Fusobacteriia;o_Fusobacteriales;f_Fusobacteriaceae	0.0221	2.07	1.49
	p_Fusobacteria;c_Fusobacteriia;o_Fusobacteriales;f_Fusobacteriaceae;g_Fusobacterium	0.0221	2.07	1.49
	p_Fusobacteria;c_Fusobacteriia;o_Fusobacteriales;f_Fusobacteriaceae;g_Fusobacterium;s_	0.0221	2.07	1.49
	p_Proteobacteria;c_Alphaproteobacteria	0.0451	0.96	1.31
	p_Proteobacteria;c_Alphaproteobacteria;o_RF32	0.0351	0.00	0.89
	p_Proteobacteria;c_Alphaproteobacteria;o_RF32;f_	0.0351	0.00	0.89
	p_Proteobacteria;c_Alphaproteobacteria;o_RF32;f_;g_	0.0351	0.00	0.89
	p_Proteobacteria;c_Alphaproteobacteria;o_RF32;f_;g_;s_	0.0351	0.00	0.89
_0105	p_Proteobacteria;c_Betaproteobacteria;o_Burkholderiales;f_Oxalobacteraceae;g_Ralstonia;s_	0.0072	0.00	1.99
	p_Proteobacteria;c_Gammaproteobacteria;o_Pasteurellales;f_Pasteurellaceae;g_Haemophilus	0.0358	1.87	1.32
	p_Proteobacteria;c_Gammaproteobacteria;o_Pasteurellales;f_Pasteurellaceae;g_Haemophilus;s_parainfluenzae	0.0358	1.87	1.32

0 max

Median of CLR-transformed abundaces

Supplementary Figure S3: The abundances of Rikenellaceae and Veilonellaceae in the ileum of HFHS and HFHS+Zn-SP mice are illustrated. The centered log-ratio (CLR)-transformed abundances are presented for each family, along with their respective ratios. In one ileum sample both Rikenellaceae and Veilonellaceae were not detected (indicated in red); therefore, this sample was not considered in ratio calculations. Statistics is according to Mann-Whitney test; **, P<0.01; ***, P<0.001; ****, P<0.0001.

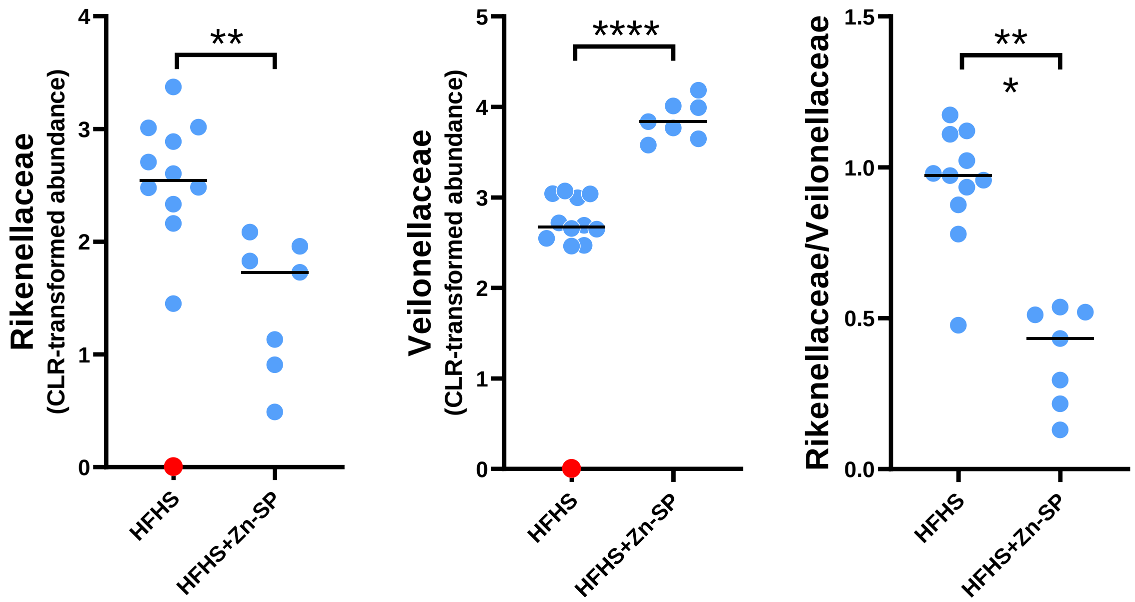

Supplement: Supplementary file 1 [file nutrients-16-00552-s001.zip › nutrients-2841101-supplementary.pdf]
